# Should the Goal for the Treatment of Soil Transmitted Helminth (STH) Infections Be Changed from Morbidity Control in Children to Community-Wide Transmission Elimination?

**DOI:** 10.1371/journal.pntd.0003897

**Published:** 2015-08-20

**Authors:** Roy M. Anderson, Hugo C. Turner, James E. Truscott, T. Déirdre Hollingsworth, Simon J. Brooker

**Affiliations:** 1 London Centre for Neglected Tropical Disease Research, Department of Infectious Disease Epidemiology, School of Public Health, St. Marys Campus, Imperial College London, London, United Kingdom; 2 Mathematics Institute, University of Warwick, Coventry, United Kingdom; 3 School of Life Sciences, University of Warwick, Coventry, United Kingdom; 4 Faculty of Infectious and Tropical Diseases, London School of Hygiene & Tropical Medicine, London, United Kingdom; Yale School of Public Health, UNITED STATES

## Introduction

Morbidity induced by infection with the major soil transmitted infections (STH—*Ascaris lumbricoides*, *Trichuris trichiura*, and hookworms) results in an estimated 5.19 million disability-adjusted life years (DALYs) [1]. The World Health Organization’s (WHO) policy for control centres on three groups, preschool aged children (pre-SAC), school-aged children (SAC), and women of child bearing age, on the basis that heavy infection in these groups will have a detrimental impact on anaemia, child growth, and development. The current WHO guidelines focus on school-aged children, both for monitoring infection and as a target for treatment, although treatment of pre-SAC and women of childbearing age is also recommended where sustainable delivery mechanisms exist, especially in areas of intense transmission [2,3]. The guidelines recommend treating SAC annually where any STH prevalence falls between 20% and 50% and twice a year where it exceeds 50% [3].

The London Declaration on Neglected Tropical Diseases in 2012 endorsed WHO goals to scale up mass drug administration (MDA) for STH, so that by 2020, 75% of the pre-SAC and SAC in need will be treated regularly [4]. Building on an existing roadmap, WHO announced an intention to meet the target [2,5,6]. Progress has been good in some areas, but less so in others. In 2012, global coverage of those in need was 37% for SAC and 29% for pre-SAC [5]. Data for the more recent years is as yet to be published by WHO [5], but a huge gain in coverage is not expected, despite increased drug donations from the pharmaceutical companies who manufacture the main anthelmintics. This is due in part to the logistical challenges in getting even donated drugs to these populations, who are often beyond “the end of the road.” At present, many countries with endemic STH infections are not availing themselves of the freely donated drugs to treat children.

We are still a long way from the 2020 target of 75%. Even if this target is reached, will it be enough to eliminate transmission and the disease arising from heavy infections with STH? If not, how should the guidelines be changed to push towards morbidity control, and ideally, the eventual elimination of transmission?

### Basing Policy on Quantitative Calculations

To answer these questions, calculations are required to assess the impact of MDA targeted at particular age groupings, especially pre-SAC and SAC, on overall transmission in communities with differing levels of infection exposure. In many areas of infectious disease epidemiology and the design of interventions, the impact of control is today assessed by simulations based on mathematical models using parameter estimates from epidemiological studies (e.g., HIV and *Plasmodium falciparum* [7,8]). The neglected tropical disease (NTD) field lags behind, in the sense of largely basing target treatment levels on discussion and consensus, without detailed calculations. Much of the basic framework for the study of the transmission dynamics of helminth infections was laid down in the 1960s and 1980s [9,10]). Rather little has been achieved since that time in model development and parameter estimation. Concomitantly, little use has been made of the insights gained from these analyses in the design of public health policy for the control of STH and schistosome infections. The tools now available can rectify this shortcoming, and they can easily be adapted to include costs and benefits as outlined in this article. They should be used to refine WHO policy on treatment to aim for a robust framework that will eliminate transmission.

### Demography and Epidemiology

**Fig 1:**
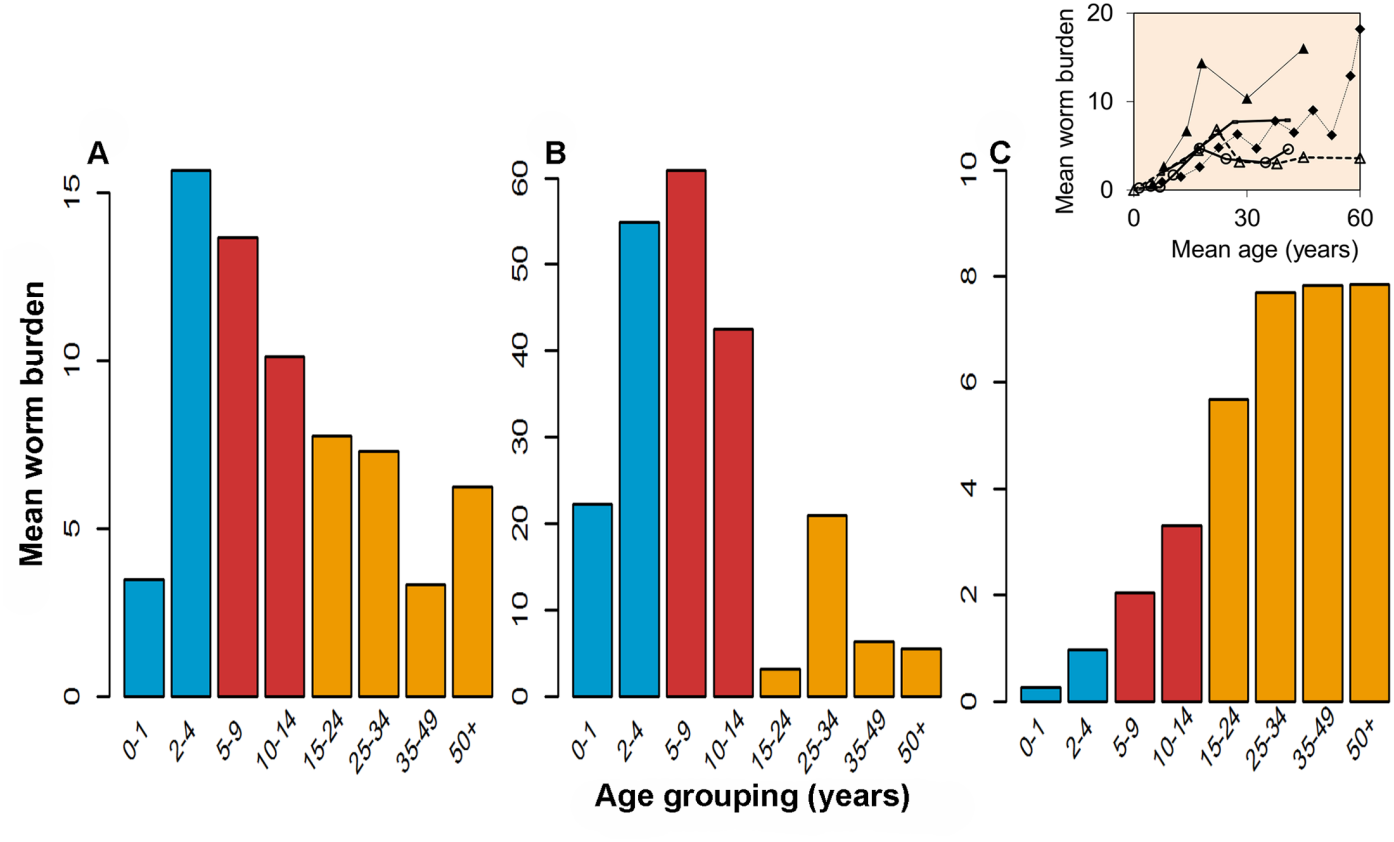
Cross-sectional surveys of the mean intensity of infection in different age groupings for *A*. *lumbricoides* (A), *T*. *trichiura* (B), and hookworm (C) based on worm expulsion studies. These are typical age intensity profiles for the three most important STH species, where the colours denote the age groupings: pre-SAC (blue), SAC (red), and adults (orange). Data for A from [25], data for B [26], and data for C [27]. The inset in Graph C represents five worm expulsion studies of *Necator americanus* showing consistent patterns in a rise in burden with age in different geographical locations [28].

A key issue concerns demography in sub-Saharan Africa and other areas with endemic infection. Typically, there are as many adults as there are pre-SAC and SAC [11]. This implies that, depending on the distribution of worms across the age classes, the adults themselves may be able to sustain transmission within a community, even when a very high fraction of the children are treated effectively. This is especially the case for hookworm where adults typically harbour the majority of worms (in some areas, more than 80% of the total population of parasites, (Fig 1—see inset in graph C)), but also applies for *Ascaris* and *Trichuris*, where up to 30% of worms and egg counts are in those older than 15 years of age [11], irrespective of the intensity of transmission in a defined area.

The impact of this substantial reservoir of infection in adults on reinfection in children can be better illuminated using deterministic and stochastic models of STH transmission, based on parameter estimates derived from cross-sectional and longitudinal worm expulsion studies and observations on demography and the mean intensity distribution across the major age groupings (pre-SAC, SAC, and adults—see Fig 1 [12,13]). A series of general conclusions emerge that support the observational studies based on demography and cross-sectional epidemiological data on age specific intensities of infection.

### Impact of Current Treatment Strategies

**Fig 2:**
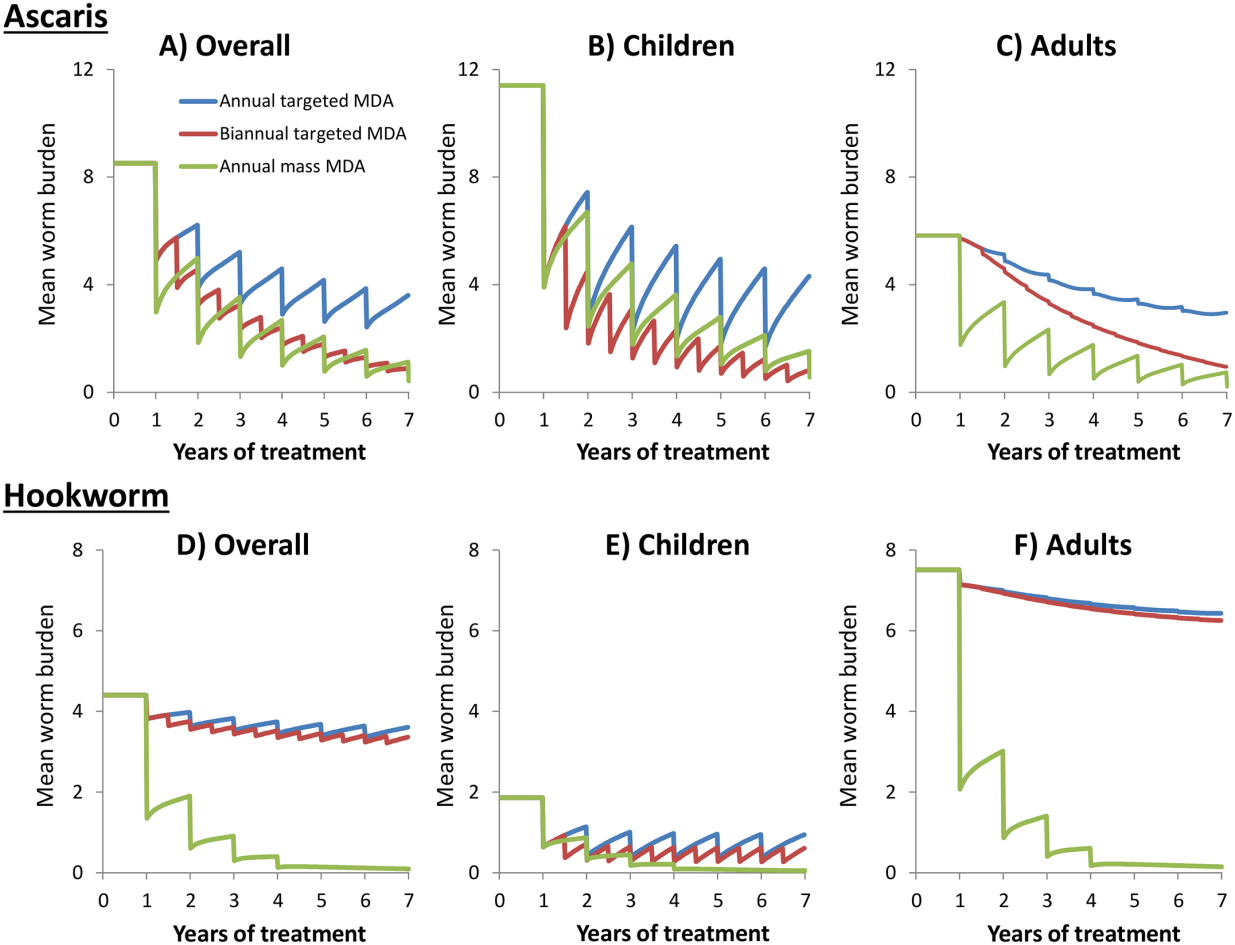
Impact of different treatment stratgies on the mean number of worms in different age groupings. The coloured lines represent different treatment strategies: green—annual community-wide MDA (75% coverage of all age groupings (pre-SAC, SAC, and adults) and 95% drug efficacy); red—biannual age group targeted MDA (pre-SAC and SAC with 75% coverage and 95% drug efficacy); and blue—annual age group targeted MDA (pre-SAC and SAC with 75% coverage and 95% drug efficacy). Graphs A–C and D–F correspond to Hookworm and *Ascaris* respectively. Graphs A and D record the overall mean number of worms across all ages. Graphs B and E record the mean number of worms in children (pre-SAC and SAC, 2–15-year-olds). Graphs C and F record the mean number of worms in adults (>15 year olds). Calculations based on a basic reproductive number, R_o_, of 2.5 (medium to high transmission setting). Model parameters described in [13].

We focus on three major issues concerning MDA, namely: who to treat, how frequently to treat, and how long to treat. In our calculations, we use a fully aged structured deterministic STH transmission model (described in [12,13]). Stochastic individual-based models for the mean worm burden give identical results to the deterministic predictions. For illustration, we compare the impact of annual and biannual treatment of pre-SAC and SAC, with annual mass treatment, in communities with *Ascaris* and hookworm. As a case study, we focus on an area of medium transmission (R_0_ values around 2–3, true prevalence (not adjusted for diagnostic sensitivity) around 70% in SAC). As illustrated in Fig 2, which records simulated MDA strategies focusing on once and twice yearly treatment of pre-SAC and SAC combined (at a coverage level of 75%), compared with community-wide treatment (at the same coverage level). Increasing the frequency of MDA in children is predicted to be marginally more effective than annual mass treatment, in terms of reducing the overall burden of *Ascaris*. In contrast, for hookworm, the analyses illustrate that increasing the frequency of MDA for pre-SAC and SAC alone has limited additional impact. Expanding the annual treatment programme to also include adults actually reduces the intensity of hookworm infections both in children and the community as a whole (Fig 2). This occurs because the adults have the majority share of the infectious reservoir (i.e., are a core group), and consequently, treating children alone does not significantly impact the level of transmission. The children get reinfected after treatment because of the reservoir in adults. This suggests that WHO guidelines to increase treatment to twice a year in high prevalence areas is unlikely to have the desired impact in areas with high hookworm prevalence. The best treatment strategy is highly dependent on the local age distribution of infection of the different STH species.

It is important to note that monitoring and evaluation programmes that only measure the impact of a school-based hookworm treatment programme in the treated age group record a much bigger impact on the children (Fig 2b) than the true impact on the entire population, as reflected by persisting worm burdens in adults (Fig 2c). School-age group-based surveillance programmes can give good estimates of the reduced morbidity in children but can lead to misleading estimates of the impact of these programmes on overall transmission in the community. A revision in the monitoring and evaluation (M & E) guidelines is required to address this problem. Current guidelines recommend that monitoring is conducted in schools, as school children are the main targets of control [14]. More recently, transmission assessment surveys (TAS) for lymphatic filariasis have been proposed as an alternative platform for monitoring STH infection [15], and the implementation of TAS in the wider community provides the opportunity to reliably track STH across a range of age classes [16].

To cross the transmission “breakpoint” [10], where transmission is eliminated, requires many years of continual treatment (Fig 2b) at moderate to high coverage (>75%), depending on the intensity of transmission (the value of R_0_). Elimination requires the treatment of adults for hookworm, and the time required to achieve this is accelerated (and required in areas of high transmission) for *Ascaris*. This conclusion also applies for *Trichuris*, and in most circumstances for the schistosome infections for which praziquantel is employed in MDA. Pre-SAC treatment for the control of morbidity induced by schistosome infections has been suggested by Stothard [17]. The practical feasibility of providing mass treatment to adults in addition to pre-SAC and SAC is demonstrated by the mass treatment campaigns for onchocerciasis and lymphatic filariasis (LF), which provide albendazole plus ivermectin or diethylcarbamazine citrate to entire communities, using community drug distributors [16]. The albendazole and ivermectin used in these programmes are also highly effective against STH [18,19], although there has been no systematic attempt to quantify the impact of the global onchocerciasis and LF control programmes on the transmission of STH despite the potential insight they afford into the impact of expanding current school-based deworming programmes.

### Costs of Different Strategies

A key issue in scaling up treatment to the whole community is cost, and this in turn depends on demography (the proportions of the population in each age grouping), who is treated, at what coverage level, how often, and for how long. If transmission is not interrupted by only focusing on pre-SAC and SAC, treatment must continue forever if no other conditions change [13]. Water, sanitation, and hygiene (WASH) programmes have the potential to radically change the picture, if they can be designed to permanently supress transmission, but progress has been limited in many areas of endemic infection [20]. Hopefully, this situation may change in the coming decades as economic growth improves in parts of Africa and Asia, leading to better sanitation and hygiene.

Under the pessimistic assumption that treating adults is twice as expensive as treating children (many studies reporting a much lower difference [21]), we calculate that the total costs of community-wide treatment over a 20 year period (discounting at 3% per year) are much less (approximately 60%) than that for repeated annual treatment of pre-SAC and SAC, given that the former has only to continue for three years (with the assumptions in Fig 2), while the latter must continue beyond the 20-year time horizon in hookworm transmission areas of intermediate intensity (Turner et al. manuscript in preparation).

### New Treatment Guidelines and a New Strategy

Our calculations suggest that the current guidelines need modification, particularly regarding the recommendation to increase treatment frequency if the prevalence of any STH is greater than 50% in SAC. Calculations suggest that in most areas where hookworm is the dominate infection, it is better to broaden treatment across all age classes instead of treating children twice a year. This leads to the more efficient use of limited resources in the longer term. For high transmission *Ascaris* areas, the best option is both increasing frequency and broadening coverage across all age classes.

Drug efficacy for *Trichuris* is known to be low with monotherapy using either Albendazole or Mebendazole (the standard treatments for STH) [22], and hence in settings where *Trichuris* is the dominant STH, alternative approaches will be required. One such approach that has been shown to notably increase the treatment efficacy against *Trichuris* is coadministering Ivermectin with the benzimidazoles or using other drug combinations [23]. With dual therapy, broadening the coverage of age groupings to include adults may also be beneficial (depending on the local age intensity profile [Fig 1]).

What are the barriers to changing the quidelines for treatment, monitoring and evaluation? The school has many advantages for treatment delivery since high treatment coverage can be achieved for regular attenders and for surveillance as schools provide a ready sampling frame. In contrast, in some settings it has proven difficult to achieve high coverage (and good surveillance) in adults for STH (although the experience with MDA for lymphatic filariasis argues that it can be achieved [24]), and the cost of treating one adult may at times be higher when compared with treating children in a school setting. Community-wide coverage also requires an increase in drug donations. Despite this, the longer term cost calculations are compelling, given that one strategy has to be continued forever while the other offers the hope of interrupting transmission permanently. On this basis, it would seem highly desirable to change the WHO guidelines, with a concomitant emphasis on education, sustainability of current WASH programmes (to reduce transmission intensity and thereby enhance the impact of MDA), communication to encourage high treatment uptake amongst adults and better integration of STH control with that of LF where community-wide coverage has been a target for some time. The coverage levels predicted to eliminate LF transmission are much less stringent than those required for hookworm or *Ascaris*, so integrated STH and LF control is desirable, but reported coverage and frequency of treatment with Albendazole for LF must be increased to stop STH transmission. A revision of the guidelines is especially desirable when hookworm is the dominant infection, since most worms are typically harboured by adults. The cross-sectional age intensity profiles for *Ascaris* and *Trichuris* (Fig 1), and for the schistosomes, suggest that in high transmission areas, infections across the community may be maintained by adults even when children are effectively treated twice a year at high coverage levels. Of course, treating the whole community will also lead to more rapid reductions in transmission. But the effect will not be as extreme as for hookworm. To achieve the 2020 goals, treatment coverage in children must be increased significantly, but in many areas reductions in morbidity, and the highly desirable goal of stopping transmission, would both be more likely and much more rapid, if coverage is broadened to encompass adults. The debate on what is the best strategy to manage STH infection should shift from morbidity control to transmission interruption. Concomitantly, there is a need to broaden the scope of research to investigate the cost-effectiveness and feasibility of alternative treatment strategies in achieving the interruption of transmission across a range of settings. Linked to any shift from age group targeting to community-wide control is the risk of enhancing selection for drug resistant strains of the parasites, where the refugium of untreated adults no longer dilutes the gene pool of those parasites exposed to selection. But the experience with community-wide control in LF programmes, where the Albendazole drug also impacts STH, suggests this concern may not materialize in practice. However, this needs careful monitoring.
